# Research on a High-Temperature Electromagnetic Ultrasonic Circumferential Guided Wave Sensor Based on Halbach Array

**DOI:** 10.3390/mi16040367

**Published:** 2025-03-24

**Authors:** Yuanxin Li, Jinjie Zhou, Jiabo Wen, Zehao Wang, Liu Li

**Affiliations:** 1 School of Mechanical Engineering, North University of China, Taiyuan 030051, China; lee1031881714@163.com (Y.L.); wen_jiabo@163.com (J.W.); 15735111531@163.com (Z.W.); ll6fan9527@163.com (L.L.); 2 Shanxi Key Laboratory of Intelligent Equipment Technology in Harsh Environment, Taiyuan 030051, China

**Keywords:** EMAT, high-temperature pipeline, defect detection, CLamb waves, Halbach array, lift-off

## Abstract

High-temperature pipelines, as core facilities in the fields of petrochemical and power, are constantly exposed to extreme working conditions ranging from 450 to 600 °C, facing risks of stress corrosion, creep damage, and other defects. Traditional shutdown inspections are time-consuming and costly. Meanwhile, existing electromagnetic acoustic transducers (EMATs) are restricted by their high-temperature tolerance (≤500 °C) and short-term stability (effective working duration < 5 min). This paper proposes a high-frequency circumferential guided wave (CLamb wave) EMAT based on a Halbach permanent magnet array. Through magnetic circuit optimization (Halbach array) and multi-layer insulation design, it enables continuous and stable detection on the surface of 600 °C pipelines for 10 min. The simulations revealed that the Halbach array increased the magnetic flux density by 1.4 times and the total displacement amplitude by 2 times at a magnet’s large lift-off (9 mm). The experimental results show that the internal temperature of the sensor remained stable below 167 °C at 600 °C. It was capable of detecting the smallest defect of a φ3 mm half-hole (depth half of the wall thickness), with a signal attenuation rate of only 0.32%/min. The signal amplitude of Q235 pipelines under high-temperature short-term detection (<5 min) was 1.5 times higher than that at room temperature. However, material degradation under high temperature led to insufficient long-term stability. This study breaks through the bottleneck of long-term detection of high-temperature EMATs, providing a new scheme for efficient online detection of high-temperature pipelines.

## 1. Introduction

As key components of the modern energy system, oil and gas transmission pipe networks and high-temperature pressure-bearing pipelines hold an irreplaceable strategic position in sectors such as power production (thermal and nuclear), regional heating, and petrochemical industries. Particularly in industries like petroleum, chemical engineering, and power generation, the application of high-temperature metal pipelines is widespread. Under typical high-temperature and high-pressure operating conditions, defects such as pitting corrosion, uniform corrosion, and stress corrosion cracking can develop both internally and externally within the pipelines, especially in areas obscured by supports or adjacent pipelines. To prevent these defects from escalating to pipe wall perforation and rupture—events that could result in significant loss of life and property—it is often necessary to halt operations, reduce temperatures, and conduct inspections. However, this process typically requires considerable time, and frequent shutdowns undoubtedly impose substantial economic losses on enterprises. The introduction of non-destructive testing (NDT) technology facilitates timely defect detection and enhances operational efficiency.

Nevertheless, commonly employed NDT techniques face significant challenges when applied to complex high-temperature pipelines. For instance, piezoelectric ultrasonic testing (PUT) is constrained by the thermal stability of the coupling agent and the piezoelectric material itself, making it difficult to ensure stable and reliable testing at elevated temperatures [1,2,3,4]. Laser ultrasonic testing (LUT), while promising, is limited by its high equipment costs and the significant impact of surface morphology on transducer efficiency, rendering it unsuitable for inspecting high-temperature pipelines [5,6,7].

Electromagnetic ultrasonic testing technology is a highly potential non-destructive testing technique applicable under high-temperature conditions. Due to the absence of the need for coupling agents during the testing process [8], it enables non-contact defect detection [9], offering characteristics such as safety and convenience. It has garnered considerable attention from numerous scholars in recent years. Electromagnetic ultrasonic testing technology is categorized into electromagnetic ultrasonic bulk wave testing and electromagnetic ultrasonic guided wave testing. Currently, electromagnetic ultrasonic bulk wave sensors have been utilized in high-temperature thickness measurement and flaw detection [10,11,12,13,14]. Despite the fact that electromagnetic ultrasonic bulk waves have demonstrated certain advantages in high-temperature defect detection, their insufficient sensitivity to internal defects, their limited propagation distance, and the requirement of stripping all coatings for point-by-point scanning and flaw detection, as well as their inability to accommodate complex working conditions, have restricted their extensive application in high-temperature pipelines. For instance, areas of high-temperature pipelines that are partially shielded by support structures or other pipelines and thus inaccessible cannot be inspected using electromagnetic ultrasonic bulk wave sensors. To overcome these limitations, scholars have gradually redirected their focus to electromagnetic ultrasonic guided wave technology. Compared to bulk waves, guided waves can propagate over longer distances within pipelines, possess a larger detection range, exhibit higher detection efficiency, and demonstrate greater sensitivity to defects on both the inner and outer walls of the pipeline. Guided waves employed for pipeline inspection are classified into axial guided waves (L mode, T mode, and F mode) and circumferential guided waves (CSH wave, Lamb wave). Axial guided wave detection technology is primarily utilized for low-frequency detection of long-distance defects in the axial direction of pipelines. Owing to its low attenuation characteristics, it holds significant advantages in the rapid screening of long-distance defects in high-temperature pipelines [15,16,17,18]. Nevertheless, due to its large wavelength, it has relatively low positioning accuracy for some minute defects. Additionally, the propagation direction of axial guided waves renders them less sensitive to circumferential defects (such as circumferential cracks and unfused welds), thereby making it challenging to achieve high-resolution imaging of circumferentially distributed local damage (such as local corrosion pits). Circumferential guided waves, given their propagation characteristics along the circumferential direction of the pipeline, constitute an ideal option for resolving this issue.

Circumferential guided wave detection technology is a simple, reliable, and effective non-destructive testing approach. Defect information on a full circle of the pipeline can be acquired through single-point excitation. Meanwhile, by scanning along the axial direction of the pipeline, the entire pipeline, including inaccessible regions, can be comprehensively examined. Merely removing a portion of the cladding layer enables complete detection of the circumferential or axial areas of the pipeline, featuring a high detection efficiency. However, the weak signal, multi-modal nature, and low operating temperature of electromagnetic ultrasonic guided wave sensors (EMATs) significantly restrict their application in severe high-temperature environments. To address these challenges, researchers have made numerous attempts. Cai et al. [19] proposed an improved single-sided one-transmitter-one-receiver EMAT. Compared with the traditional EMAT, the optimized design enhanced the horizontal static magnetic field excitation, significantly increasing the transduction efficiency of the sensor. Rieger et al. [20] designed a compact and small-sized EMAT, eliminating the overly large permanent magnet and consisting solely of two closely adjacent slender and flat coils. It is capable of unidirectionally transmitting and receiving ultrasonic Lamb waves in 1 mm thick steel plates. Yang et al. [21] presented an EMAT structure with a periodic magnet configuration. By augmenting the flux density of the local magnetic field, the amplitude of the S0 mode Lamb wave was strengthened. On this basis, an improved EMAT was proposed. Compared with the traditional EMAT, the improved EMAT can increase the amplitude of the Lamb wave S0 mode by seven times. Liu et al. [22] put forward a novel focused EMAT design with a narrow magnet, which can both enhance the A0 mode signal of the ultrasonic Lamb wave and correct the distorted waveform. In contrast to the traditional EMAT, the new EMAT signal does not distort, and the amplitude of the A0 mode Lamb wave also increases. Dhayalan et al. [23] proposed a new method utilizing a soft magnetic alloy strip (MFC) as a flux concentrator to enhance the amplitude of the EMAT ultrasonic signal. Experiments demonstrated that the peak signal amplitude with MFC was twice that without MFC. Ren et al. [24] introduced silicon steel laminations as the backplane into the electromagnetic acoustic transducer (EMAT). Through experiments, it was proven that adding silicon steel laminations can increase the dynamic magnetic field of the EMAT coil and the size of the eddy current on the sample surface, thereby enhancing the transduction efficiency of the EMAT. Liu et al. [25] proposed an EMAT that uses a magnetic concentrator to regulate the mode of the excitation signal. By replacing the magnetic concentrator and thereby controlling the center distance of the static magnetic field of the magnet, the mode of the generated signal can be controlled. Kogia et al. [26] developed a pair of water-cooled EMATs, capable of exciting and receiving SH waves on a flat plate at a maximum temperature of 500 °C. However, the detection time is very short, and there is no study on defect detection ability at high temperatures.

The typical service temperature range of industrial high-temperature pipelines (450–600 °C) has significantly surpassed the applicable temperature ceiling of the existing electromagnetic ultrasonic guided wave detection technology (EMAT) (≤500 °C). More importantly, when conducting pipeline flaw detection, a continuous and stable scanning for at least 10 min is necessary. However, the effective working duration of the EMAT devices reported in the current literature under 600 °C conditions is generally less than 5 min. Therefore, the development of a high-temperature EMAT with high-temperature tolerance (600 °C surface contact) and long-term stability (stable signal within a 10-min detection cycle) not only fills the adaptability gap of the existing technologies under high-parameter working conditions but also provides reliable online detection solutions for industries such as petrochemical and power, which holds significant engineering value for ensuring the safe operation of high-temperature pressure pipelines.

To improve the high-temperature tolerance and long-term stability of EMAT and increase the signal amplitude of EMAT at large lift-off, a high-frequency and high-temperature CLamb wave EMAT based on a Halbach array with large magnet lift-off was developed in this paper. The structure of this paper is as follows: The second part introduces the basic principle of EMAT, compares the lift-off performance of traditional magnets and Halbach magnets through simulation and experiments, and develops a high-frequency and high-temperature CLamb wave EMAT; the third part establishes a high-temperature experimental platform; the fourth part experimentally validates the defect detection capability of EMAT on different materials at high temperatures; and the fifth part conducts a comprehensive summary of the experimental results.

## 2. EMAT Design

**Figure 1:**
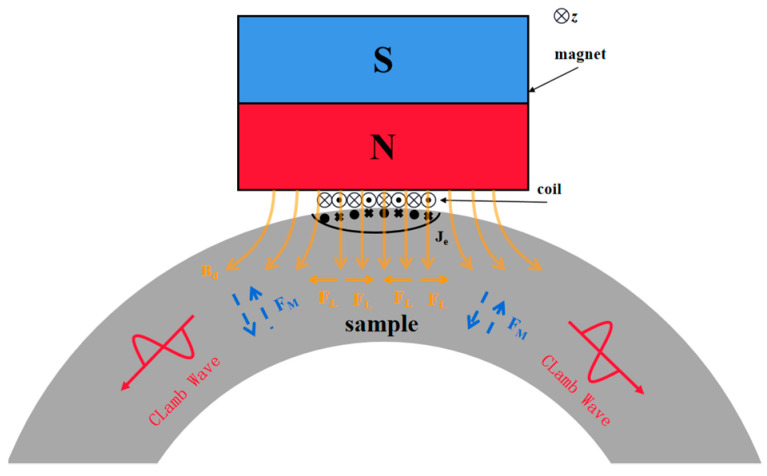
Mechanism of EMAT.

EMAT is generally composed of magnets (permanent magnets or electromagnets) and coils, and the principal objects of its application are metallic materials possessing electrical conductivity or magnetic permeability [27]. The mechanism of action is depicted in Figure 1. When EMAT is excited, an alternating current is required to be passed through the coil, and the varying current generates a dynamic magnetic field. This dynamic magnetic field induces eddy currents on the upper surface of the material. The charged particles within the eddy current field are influenced by the magnetic field, thereby generating Lorentz force. The grains vibrate reciprocally under the force, forming ultrasonic waves that propagate directionally. Its value is equal to the product of the eddy current density and the total magnetic field, as indicated in Equation (1):

(1) $$F_{L}=F_{s}+F_{d}=J_{e}\times \left(B_{s}+B_{d}\right)$$

Among them, *F_L_* represents the Lorentz force, *F_s_* represents the static Lorentz force, *F_d_* represents the dynamic Lorentz force, *J_e_* denotes the eddy current induced by the coil, *B_s_* indicates the static magnetic field, and *B_d_* refers to the dynamic magnetic field. It can be observed that the Lorentz force has a linear relationship with the magnitude of the alternating current and the intensities of the static/dynamic magnetic fields. It increases as the current/magnetic field increases, and vice versa. When detecting ferromagnetic samples, the magnetostrictive force [27] also needs to be taken into account. Under free pressure conditions, the magnetostrictive force can be defined as presented in Equation (2):

(2) $$F_{M}=-\nabla \left(e^{T}H_{k}\right)$$

**Figure 2:**
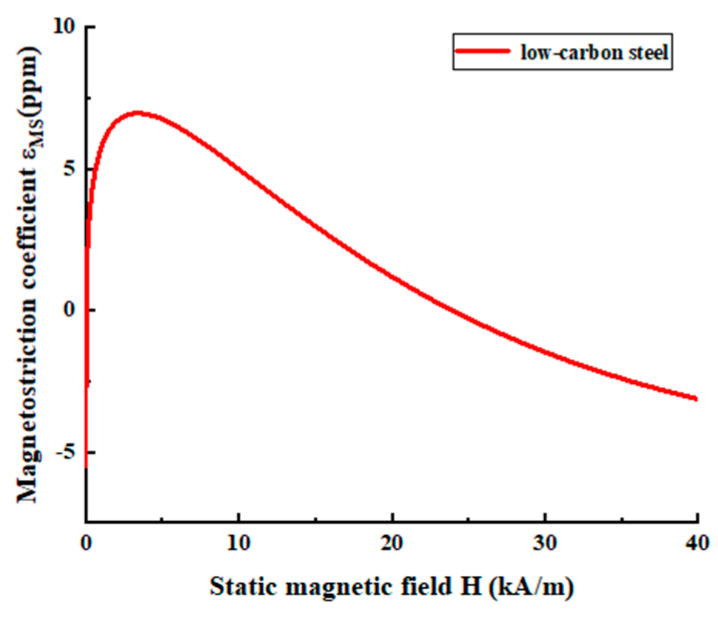
Magnetostrictive curve of low-carbon steel.

Among them, *F_M_* represents the Magnetostrictive force, *H_k_* is the dynamic magnetic field matrix, and *e^T^* is the inverse magnetostrictive matrix. The inverse magnetostrictive matrix is related to the magnetostrictive coefficient of the sample and the magnetic field intensity it is subjected to. Unlike the Lorentz force, the magnetostrictive force has a nonlinear relationship with the external magnetic field intensity. The specific variation trend requires analysis through the magnetostrictive curve of the specimen and the magnitude of the static magnetic field [28]. The magnetostrictive curve of low-carbon steel is depicted in Figure 2 [28]. It can be observed that the magnetostrictive coefficient does not increase continuously with the increase in the magnetic field intensity. In ferromagnetic materials, there is not merely the magnetostrictive mechanism but also a magnetization force mechanism. Since, in ferromagnetic materials, the magnitude of the magnetization force is related to the size of the surface eddy current, and due to the small eddy current resulting in a relatively small force, which is several orders of magnitude lower than the other two forces, it is often ignored.

The coil configuration and magnet arrangement determine the waveform mode of the EMAT. The combination of cube-shaped permanent magnets and folded coils on the plate can excite/receive Lamb waves, and on the pipe, it can excite/receive CLamb waves. This type of EMAT is dominated by the Lorentz force at room temperature [29,30,31]. However, at high temperatures, the magnetostrictive force gradually becomes the dominant force in similar EMATs on ferromagnetic materials [32]. This study focuses on the performance of EMATs on ferromagnetic materials (low-carbon steel, ferritic alloy steel).

In this study, the coils were fabricated using the printed circuit board method. To prevent the coil substrate from melting and deforming at high temperatures, causing short circuits, the coils were encapsulated with high-temperature ceramic adhesive and polyimide film. Samarium cobalt permanent magnets with a maximum operating temperature of 350 °C were selected. When the operating temperature is above 180 °C, their maximum magnetic energy product, coercivity, temperature stability, and chemical stability all exceed those of commonly used neodymium iron boron permanent magnets.

Since the maximum operating temperature of both the coil and the magnet cannot exceed 350 °C, it is necessary to consider setting an insulating layer between the magnet and the coil. However, EMAT is usually very sensitive to the distance between the sample and itself, where the distance between the magnet and the sample is called the magnet lift-off, and the distance between the coil and the sample is called the coil lift-off. An increase in the magnet lift-off will reduce the magnetic field strength on the surface of the pipe, and an increase in the coil lift-off will reduce the eddy current induced on the surface of the pipe. Therefore, the larger the magnet lift-off/coil lift-off, the smaller the amplitude of the signal generated by the Lorentz force. The magnetostrictive force, due to its nonlinear relationship with the static magnetic field, requires specific analysis of its changing trend. Therefore, the lift-off performance of different magnets still needs to be studied through simulation and experiments.

**Figure 3:**
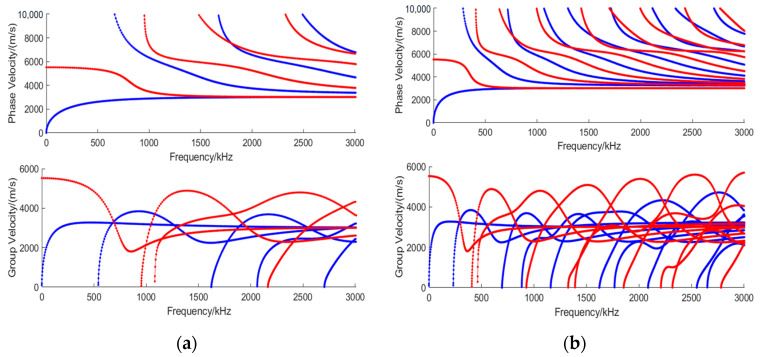
Lamb wave dispersion curves, the red line indicates S mode and the blue line indicates A mode: (**a**) 3 mm iron plate, (**b**) 7 mm iron plate.

In actual experiments, the optimal excitation frequency point also needs to be determined based on the dispersion curve of Lamb waves. The dispersion curves of Lamb waves are shown in Figure 3a,b, where the symmetric mode (S mode) is represented by the red curve and the asymmetric mode (A mode) is represented by the blue curve. It can be seen that regardless of which frequency point is selected, at least two Lamb wave modes will be generated, and the higher the selected frequency and the thicker the wall thickness, the more modes will be excited, which increases the difficulty of detecting high-frequency Lamb waves. When Lamb waves are excited at an inappropriate frequency at high frequencies, many wave packets will be generated simultaneously, causing signal aliasing and making defect identification very difficult. However, at certain specific frequencies, due to the almost identical group velocities between adjacent modes, they will superimpose to form unique mode clusters. These mode clusters with shorter wavelengths have higher resolution and sensitivity, making them suitable for identifying small pinhole defects. Therefore, the selection of appropriate Lamb modes and specific working points should be considered.

### 2.1. Finite Element Simulation

**Figure 4:**
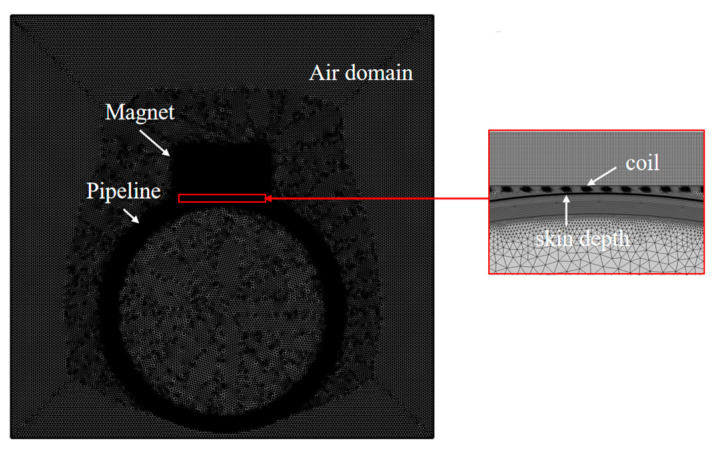
Simulation model.

To compare the lift-off performance of the Halbach array magnet EMAT and the conventional magnet EMAT on ferromagnetic pipes, finite element simulation was conducted using COMSOL Multiphysics 5.6 software. COMSOL Multiphysics 5.6 software includes physical field interfaces such as AC/DC and structural mechanics, which can apply multiphysics field coupling to the test piece. It can achieve the simulation of a single Lorentz force mechanism model and a single magnetostrictive mechanism model. Therefore, COMSOL software was used in this section to complete the modeling and simulation. The two-dimensional finite element simulation geometry model established in COMSOL is shown in Figure 4. This model consisted of permanent magnets, high-frequency coils, pipes, and air domains. The size of the conventional magnet was 30 mm wide and 30 mm high. The Halbach magnet array was 60 mm wide and 30 mm high, with the main magnet having the same size as the conventional magnet and the secondary magnet being 7.5 mm wide and 30 mm high. The high-frequency coil was represented by 12 rectangles, with a coil width of 0.5 mm; a thickness of 0.05 mm; and a coil spacing of half the wavelength, 2.5 mm. The outer diameter of the pipe was 165 mm, and the thickness was 3 mm. The bottom of the magnet was 1 mm away from the pipe and 0.5 mm away from the coil, and the coil was 0.5 mm away from the pipe surface. Due to the high-frequency alternating current excitation, a high-frequency changing magnetic field was generated, which would produce a high-frequency changing eddy current within the skin depth of the test piece. The generated eddy current had the same frequency as the excitation current but in the opposite direction. To increase the calculation accuracy, smaller quadrilateral meshes were generated within the skin depth of the pipe.

The simulation model established in this study encompasses one solid mechanics physical field and two magnetic fields physical fields. The solid mechanics physical field was primarily employed to calculate the ultrasonic displacement value generated by the Lorentz force/magnetostrictive force in the specimen. The first magnetic field was utilized to compute the static magnetic field provided by the permanent magnet, and the second magnetic field was adopted to calculate the dynamic magnetic field generated by the coil. The residual magnetic flux density of the permanent magnets was uniformly 1.21 T, and the excitation current was a five-cycle sine wave modulated by a Hanning window. The current directions of adjacent coils were opposite, with a frequency of 520 kHz, determined by the intersection point of the dispersion curve and the wavelength, and a magnitude of 10 A. The material of the pipe was set as low-carbon steel, and the material of the coil was set as copper. The parameters of the relevant materials utilized in the simulation are presented in Table 1.

**Figure 5:**
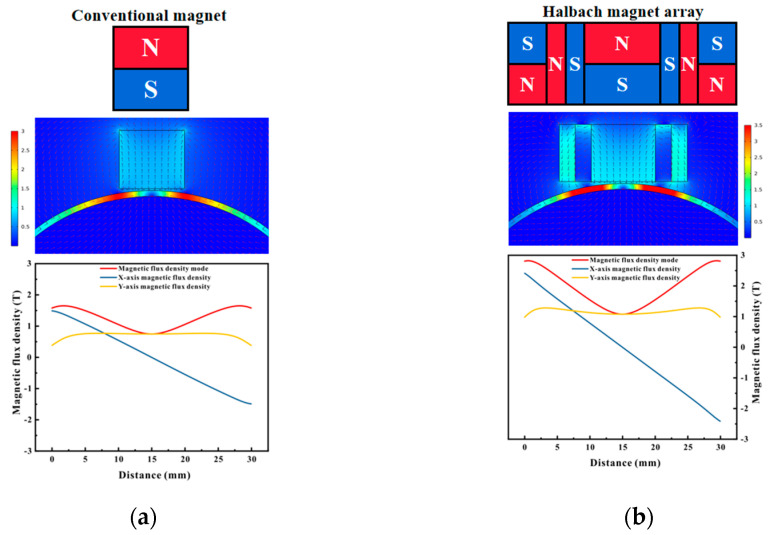
Magnet configuration and magnetic flux density distribution: (**a**) conventional magnet; (**b**) Halbach magnet.

Figure 5 presents the configuration diagrams of the conventional EMAT and the Halbach EMAT, along with the distribution of magnetic flux density on the pipe surface. The horizontal and vertical directions are defined as the x-axis and the y-axis, respectively. It can be observed from Figure 5 that in the central working area of the permanent magnet, the magnetic flux density in the vertical direction predominated, while at both ends, the magnetic flux density in the horizontal direction predominated, and the directions were opposite. Compared with the traditional magnet, the magnetic flux density on the pipe surface of the EMAT with the Halbach magnet array was approximately 1.4 times higher.

**Figure 6:**
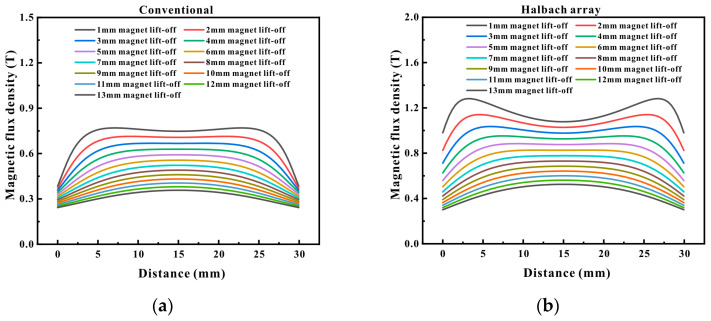
Magnetic flux density distribution at different lift-offs: (**a**) conventional magnet; (**b**) Halbach magnet.

When EMAT was employed for detection on ferromagnetic materials, the Lorentz force and the magnetostrictive force were the principal forces. These two forces were influenced by the magnitude of the eddy current induced on the pipe surface and the intensity of the static magnetic field. The distance between the magnet in EMAT and the specimen is termed as the magnet lift-off, and the distance between the coil and the specimen is referred to as the coil lift-off. As the magnet lift-off increased, the magnetic field strength on the pipe surface reduced, and as the coil lift-off increased, the eddy current induced on the pipe surface diminished. Consequently, the greater the magnet lift-off/coil lift-off, the smaller the signal amplitude produced by the Lorentz force. However, due to the nonlinear relationship between the magnetostrictive force and the static magnetic field, its variation trend requires specific analysis. To investigate the differences, Figure 6 depicts the variation of magnetic flux density under different magnet lift-offs, commencing from a 1 mm magnet lift-off and incrementing the lift-off distance by 1 mm in steps. It can be conspicuously observed that regardless of the magnet lift-off, the magnetic flux density generated by the Halbach magnet on the pipe surface was consistently larger than that of the conventional magnet. When the magnet lift-off was 1 mm, the magnetic flux density on the pipe surface directly beneath the conventional magnet was approximately 0.74 T, while that directly beneath the Halbach magnet was approximately 1.07 T. As the lift-off distance escalated, the magnetic flux density steadily decreased. When the lift-off distance was 7 mm, the magnetic flux density of the conventional magnet reduced to approximately 0.52 T, and that of the Halbach magnet decreased to approximately 0.72 T. When the lift-off distance was 13 mm, the magnetic flux density of the conventional magnet decreased to approximately 0.35 T, while that of the Halbach magnet reduced to approximately 0.49 T. The magnetic flux density of the Halbach magnet at a 7 mm magnet lift-off was comparable to that of the conventional magnet at a 1 mm lift-off.

**Figure 7:**
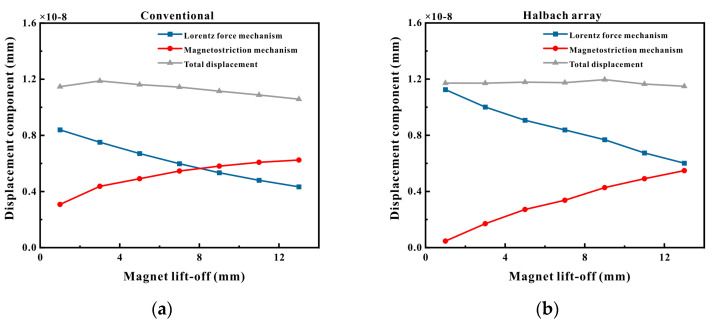
Displacement at different magnetic lift-offs: (**a**) conventional magnet; (**b**) Halbach magnet.

To conduct a comparison between the magnitudes of ultrasonic waves produced by Halbach magnets and conventional magnets under diverse mechanisms and at varying lift-offs, the particle displacement values generated within the pipeline were extracted for contrast. The observation point was chosen to be at a distance of 100 mm from the excitation location. The vertical displacements at the observation point for different magnet lift-offs are depicted in Figure 7a,b.

It is conspicuously observable from Figure 7a that for conventional magnets, as the lift-off distance escalated, the displacement engendered by the Lorentz force steadily declined, while the displacement produced by the magnetostrictive force consistently increased. The total displacement attained its maximum when the magnet lift-off was 2 mm and subsequently decreased as the lift-off distance augmented. When the magnet lift-off was 1 mm, the displacement resulting from the Lorentz force mechanism was approximately 2.72 times that of the magnetostrictive mechanism, signifying that the Lorentz force mechanism held a dominant position. As the lift-off distance gradually expanded, when the magnet lift-off exceeded 7 mm, the magnetostrictive mechanism assumed dominance. When the magnet lift-off reached 13 mm, the displacement generated by the magnetostrictive force was approximately 1.43 times that of the Lorentz force.

From Figure 7b, it can be discerned that for Halbach magnets, the changing tendencies of the displacements generated by the Lorentz force and the magnetostrictive force with the increase in the lift-off distance were identical to those of conventional magnets. Nevertheless, the total displacement reached its peak when the magnet lift-off was 9 mm and then diminished as the lift-off distance increased. Furthermore, when the magnet lift-off was 1 mm, the displacement produced by the Lorentz force mechanism was approximately 24.3 times that of the magnetostrictive mechanism, indicating that the Lorentz force mechanism was decidedly dominant. As the lift-off distance gradually enlarged, when the magnet lift-off was 13 mm, the displacements generated by the magnetostrictive mechanism and the Lorentz force mechanism were approximately equivalent.

**Figure 8:**
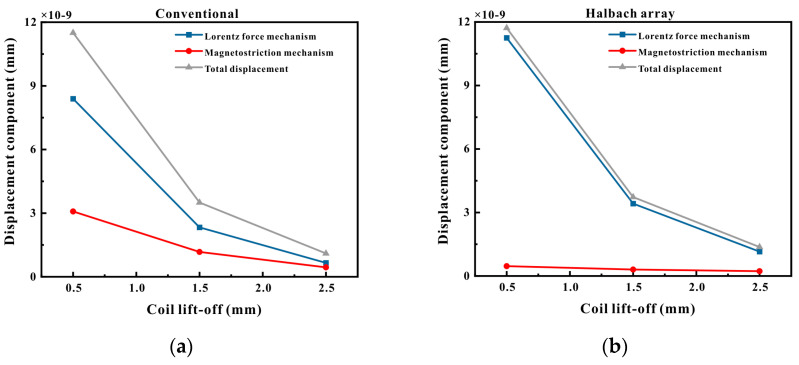
Displacement at different coil lift-offs: (**a**) conventional magnet; (**b**) Halbach magnet.

While maintaining the magnet lift-off at 1 mm invariant, the coil lift-off was varied to extract the vertical displacements at the observation point under different coil lift-offs, as depicted in Figure 8a,b. It is conspicuously evident from Figure 8a that for the conventional magnet, as the coil lift-off distance increased, the displacements produced by both the Lorentz force and the magnetostrictive force steadily decreased, and the attenuation of the ultrasonic wave by the Lorentz force mechanism was significantly greater than that of the magnetostrictive force mechanism. As the coil lift-off distance gradually enlarged, the magnitudes of the two asymptotically approached each other. When the coil lift-off distance was 2.5 mm, the displacement generated by the Lorentz force mechanism was approximately 1.47 times that generated by the magnetostrictive force. From Figure 8b, it can be observed that for the Halbach magnet, as the coil lift-off distance increased, the variation trends of the displacements generated by the Lorentz force and the magnetostrictive force were identical to those of the conventional magnet. The attenuation of the ultrasonic wave by the Lorentz force mechanism was likewise significantly greater than that of the magnetostrictive force mechanism. When the coil lift-off distance was 2.5 mm, the displacement generated by the Lorentz force mechanism was approximately 5.02 times that generated by the magnetostrictive force.

The simulation results indicate that Halbach magnets possess a higher magnetic field intensity than conventional magnets. When operating on ferromagnetic pipes, they generated a larger Lorentz force and a smaller magnetostrictive force, with the total displacement being similar to that of conventional magnets. Nevertheless, when the lift-off of the large Halbach magnet was significant, the total displacement it generated was greater than that of the conventional magnet, and its amplitude still increased when the magnet lift-off increased to 9 mm. When the coil lift-off was augmented, the total displacement produced by the Halbach magnet was likewise larger than that of the conventional magnet.

### 2.2. Lift-Off Experiment

**Figure 9:**
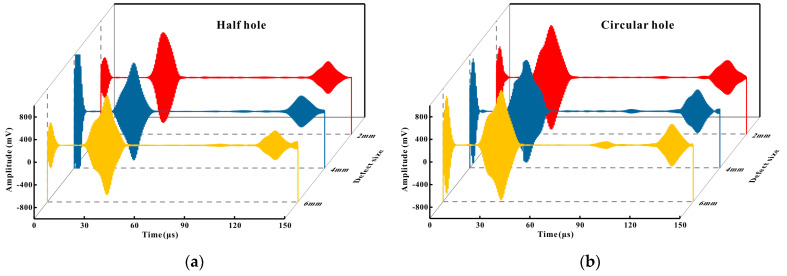

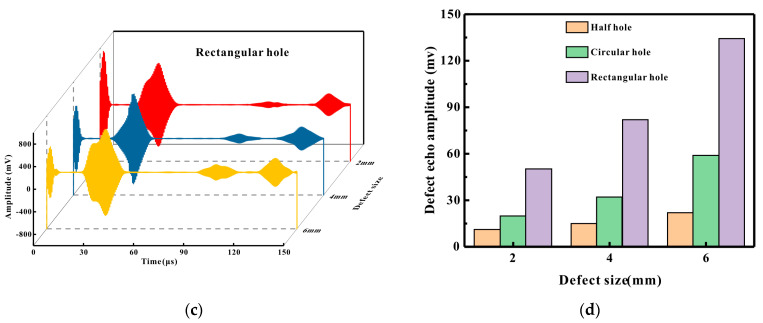
Comparison of defect signals of different sizes: (**a**) half hole; (**b**) circular hole; (**c**) rectangular hole; (**d**) defect signals of different shape.

After simulating the lift-off performance of Halbach magnets and conventional magnets on ferromagnetic pipes in the previous section, experiments were conducted under the same conditions. The sensor used in the experiment was of the same size and structure as the transducer used in the finite element simulation in the previous section. It was composed of a cubic magnet and a meander line coil, with one exciter and one receiver arranged circumferentially along the pipe. The initial lift-off of the magnets and coils was the same as in the simulation. The pipe material was low-carbon steel (Q235), with an outer diameter of 165 mm, an inner diameter of 159 mm, and a thickness of 3 mm. Along the pipe’s axial direction, there were nine defects, including half holes, circular holes, and rectangular holes of 2 mm, 4 mm, and 6 mm, respectively. The detection results of the conventional EMAT are shown in Figure 9. From left to right in Figure 9a–c, the three wave packets were the near-end direct wave, defect echo, and far-end direct wave, respectively. It can be seen that when detecting rectangular holes and circular holes, the defect echoes of the conventional EMAT were clearly visible. However, when detecting half holes, as shown in Figure 9d, the amplitude of the defect echo was reduced to approximately 50% of that of the circular holes.

The reason why the amplitude of the crack defect echo of the same size was larger than that of the circular hole defect echo may be that when the CLamb wave met the crack defect, because the reflecting surface was flat, the reflection direction and diffraction direction of the wave were concentrated, and the echo energy was concentrated, so the received defect echo energy was strong. When the circular hole defect was encountered, when the CLamb wave interacted with the defect, because the reflecting surface was curved, the propagation direction of reflection and diffraction was more chaotic than that of the plane, and the echo energy was more dispersed, so the amplitude of the defect back wave was smaller.

**Figure 10:**
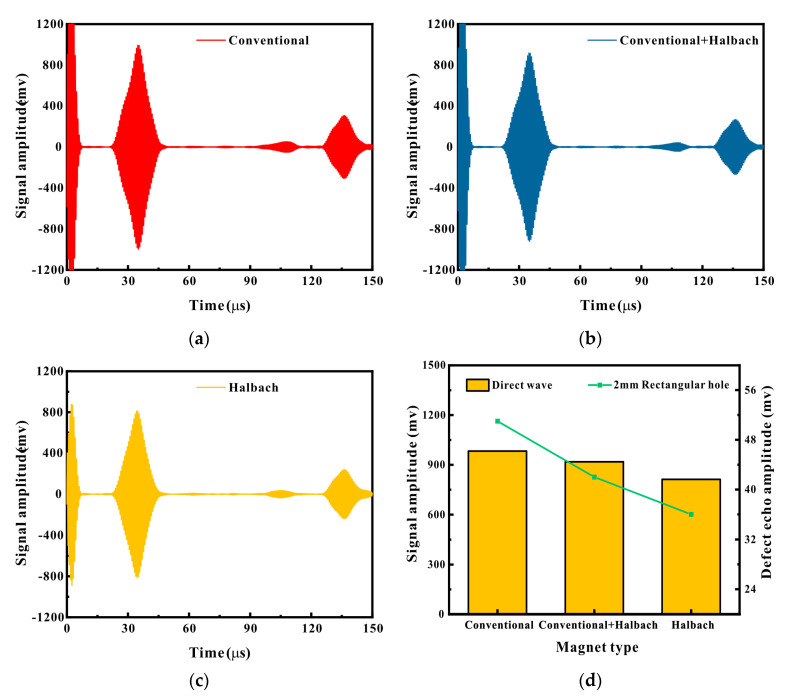
Comparison of signals from different magnet configurations: (**a**) conventional magnet; (**b**) conventional + Halbach; (**c**) Halbach magnet; (**d**) change trend of different magnets.

The comparison results of defect detection capabilities of EMATs with different magnet configurations are presented in Figure 10. It can be observed that at the initial lift-off, the EMAT signal was the greatest when both the excitation and receiving sensors employed conventional magnets. The signal was secondarily large when the excitation sensor utilized a conventional magnet and the receiving sensor adopted a Halbach magnet. Nevertheless, when both the excitation and receiving sensors utilized Halbach magnets, the signal was the smallest, with the amplitude of the direct wave being merely 83% of that of the conventional magnet and the amplitude of the defect echo being only 72% of that of the conventional magnet. It is hypothesized that this situation is associated with the nonlinearity of the magnetostrictive force.

**Figure 11:**
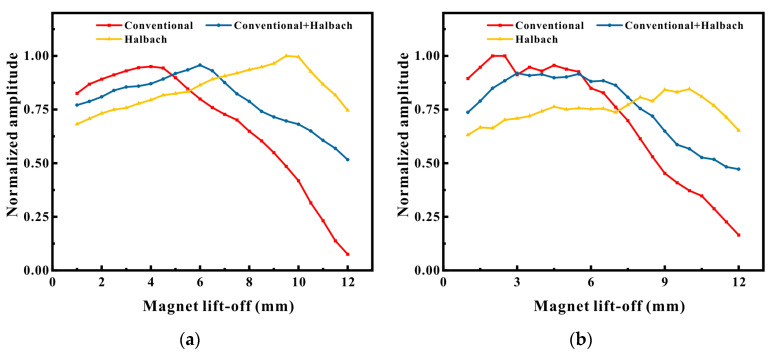

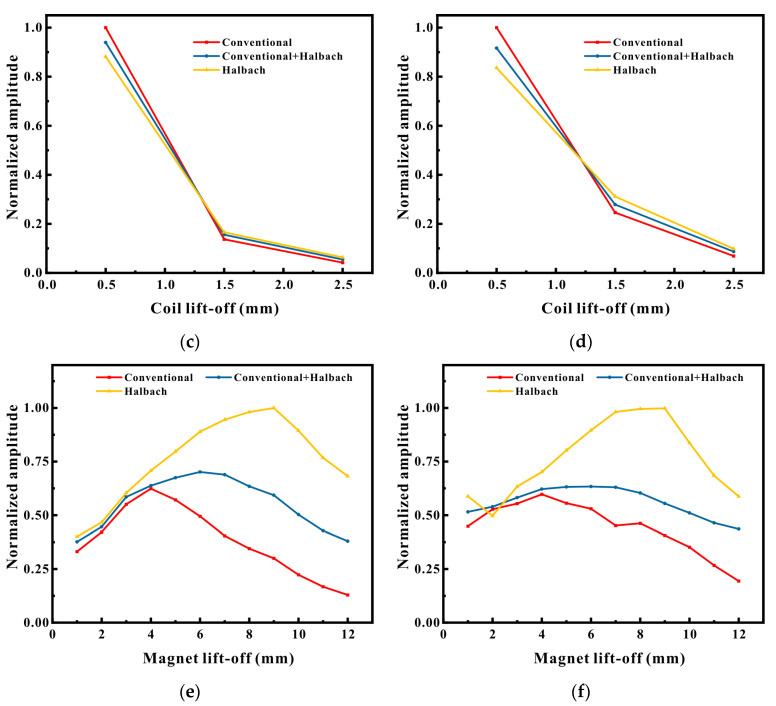
Signal variations at different lift-offs: (**a**) direct wave; (**b**) defect echo; (**c**) direct wave; (**d**) defect echo; (**e**) direct wave; (**f**) defect echo.

The lift-off performance experimental outcomes of EMATs with three distinct magnet configurations are depicted in Figure 11. From Figure 11a,b, it can be noted that at the initial lift-off, the EMAT signal amplitude with Halbach magnets was the smallest. However, as the magnet lift-off increased to 4 mm, the amplitudes of the direct wave signal and the defect echo signal of the conventional magnet reached their peaks and then commenced to decrease as the lift-off increased. The amplitude peak of the configuration with a conventional magnet and a Halbach magnet emerged between 5.5 and 6 mm. The EMAT with the Halbach magnet configuration continuously ascended with the increase in lift-off until it was between 9.5 and 10 mm. At this point, the amplitude of the direct wave of the Halbach configuration was approximately 2.06 times that of the conventional magnet, and the amplitude of the defect echo was approximately 1.92 times that of the conventional magnet. However, from Figure 11c,d, it can be perceived that as the coil lift-off increased to the maximum, the signals of any configuration of EMATs declined sharply. But the EMAT with the Halbach configuration decreased the most slowly, and when the coil lift-off was 1 mm, the signal amplitude even surpassed that of the conventional magnet. Figure 11e,f are the experimental results of magnet lift-off based on a coil lift-off of 1 mm, and the trend of variation is similar to that in Figure 11a,b.

The experimental results demonstrate that when conducting small lift-off detection on ferromagnetic materials, it is not as commonly believed that the stronger the magnetic field strength of the magnet, the better the signal. Instead, specific analysis is necessary. This corresponds to the simulation results in the previous section, indicating that the Halbach EMAT possesses superior lift-off performance compared to the conventional EMAT and has a larger signal amplitude at large lift-off.

### 2.3. High-Temperature Sensor Structure

**Figure 12:**
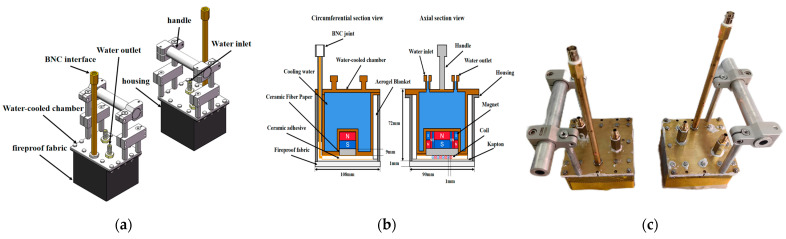
(**a**) Three-dimensional design drawing of EMAT; (**b**) two-dimensional cross-sectional view of EMAT; (**c**) physical picture of EMAT.

Figure 12a–c exhibit the final structure of the EMAT, where Figure 12c discloses the internal configuration through a bidirectional cross-sectional view. The main housing was fabricated from stainless steel, while the BNC connector, water-cooling chamber, and water inlet/outlet interfaces were made of brass. The handle was composed of aluminum alloy to reduce the overall weight of the sensor. The permanent magnet was made of Sm2Co17 material, with a residual flux density of approximately 1.2 T. The geometric dimensions of a single cubic magnet (length × width × height) were 30 mm × 30 mm × 30 mm. The Halbach magnet array was formed by bonding one cubic magnet with two 30 mm × 7.5 mm × 30 mm cubic magnets and two 30 mm × 30 mm × 7.5 mm cubic magnets using high-temperature metal adhesive under the clamping of a fixture. The meander coil was a double-layer PCB coil with a length of 30 mm, a width of 30 mm, and 30 turns, and the distance between two adjacent wires was 1 mm. The 0.2 mm thick meander coil was encapsulated in two polyimide films with high-temperature ceramic adhesive, and the thickness after encapsulation was controlled within 0.5 mm. The thermal insulation design adopted a multilayer structure: a 10 mm thick nano-aerogel blanket was placed between the stainless steel housing and the water-cooling chamber, which is renowned for its ultra-low thermal conductivity of 0.015 W/(m·K) at 25 °C, effectively blocking the heat transfer from the housing to the water-cooling chamber. A 9 mm thick ceramic fiber paper was set at the contact interface between the coil and the magnet, which had a thermal conductivity of 0.1–0.2 W/(m·K) at 25 °C, significantly retarding the heat transfer from the coil to the magnet. A dedicated groove was designed at the bottom of the water-cooling chamber to fix the permanent magnet, achieving five-sided contact heat dissipation. To address the issue of heat dissipation affected by the gap between the coil and the bottom of the water-cooling chamber, copper foil was added to fill the gap and enhance the heat conduction efficiency. An additional thermal insulation and wear-resistant layer was added at the bottom of the coil. To enable the bottom surface of the EMAT to better adapt to the curvature of the pipeline, a fire-resistant fabric (flexible material) was employed as the bottom. The coil leads extended to the BNC connector at the top of the handle, and this layout kept the BNC connector away from the heat source, ensuring that the operating temperature was below the limit.

## 3. High-Temperature Experiment Setting

**Figure 13:**
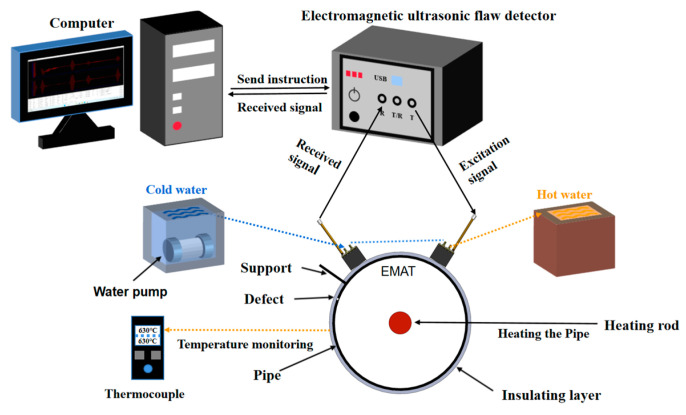
Schematic diagram of the experiment.

As depicted in the schematic diagram of the experimental setup in Figure 13, the test pipeline was composed of two materials, namely, P91 and Q235. Specifically, the P91 high-temperature pipeline had an outer diameter of 168 mm and a wall thickness of 7 mm; the Q235 pipeline had an outer diameter of 165 mm and a wall thickness of 3 mm. The experiment adopted a single excitation-single reception mode, and a 3 mm thick support structure was set in the pipeline system. The defect setting scheme was as follows: The P91 pipeline included: 1. Full penetration defects: one φ5 circular hole and one φ3 circular hole. 2. Half penetration defects: one φ5 semi-circular hole and one φ3 semi-circular hole. The Q235 pipeline included: 1. Rectangle defects: one 2 mm × 2 mm, one 4 mm × 2 mm, and one 6 mm × 2 mm through-hole defect. 2. Circular defects: one φ2 mm, one φ4 mm, and one φ6 mm through-hole defect. One φ2 mm, one φ4 mm, and one φ6 mm semi-hole defect. Through the differentiation of defect sizes (full penetration/half penetration, circular/rectangle), the study on the minimum detection capability of EMAT at high temperatures was conducted.

**Figure 14:**
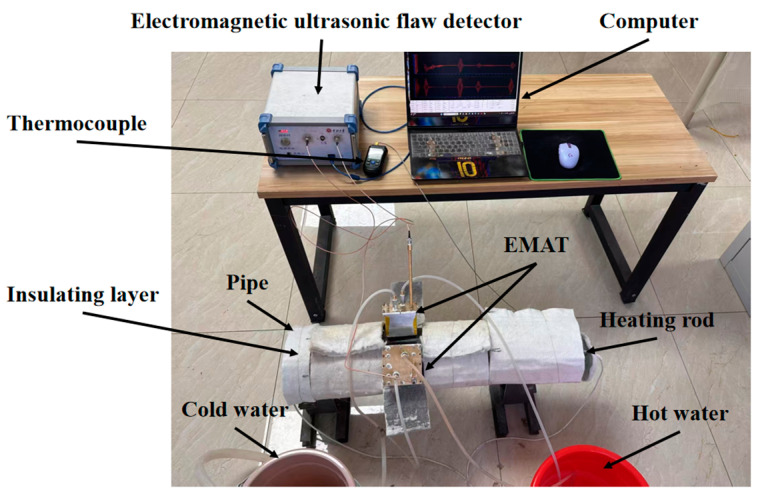
Experimental site diagram.

The configuration of the experimental system and the detection process are depicted in Figure 14. The detection platform comprised a high-temperature EMAT sensor (including the excitation end and the receiving end), an upper computer, an electromagnetic ultrasonic testing instrument, a thermal control module (consisting of a tubular heater, a thermocouple array, and a power regulator), and a circulating water-cooling system. During the detection process, the sensor was distributed along the circumference of the tube body and connected to the excitation output end and the signal receiving end of the detector, respectively, through the BNC interface. The excitation sensor was driven by a five-period Hanning window modulated signal, which was fed into the electromagnetic ultrasonic flaw detector by the computer and provided after modulation by the electromagnetic ultrasonic detector. After receiving the signal by the receiving sensor, the data were processed by the electromagnetic ultrasonic detector and finally fed back to the upper computer. The upper computer was responsible for parameter setting (including excitation frequency, excitation voltage, gain regulation, etc.), signal storage, and data analysis of the detector. The excitation frequency was 1.56 Mhz according to the intersection of the wavelength and dispersion curve, and the excitation voltage was 250 V. The original time-domain signal stored by the host computer was processed by the Gaussian filtering method, and the defect information of the pipeline was analyzed by time-domain waveform characteristics.

The elastic parameters of P91 and Q235, such as the elastic modulus and density, will decline with the increase in temperature, while the Poisson’s ratio μ will increase, resulting in a decrease in the group velocity of the CLamb wave. This leads to the delay of the signal received by the EMAT. Hence, the temperature of the pipeline should be maintained uniform and stable during the detection process. To achieve this, the thermal control module adopted a real-time control strategy: an internally suspended tubular heater was used to achieve uniform heating of the pipe body, and a 10 mm thick silica aerogel insulation layer (thermal conductivity ≤ 0.02 W/m·K) was utilized to construct the insulation layer. The temperature distribution of the pipe wall was monitored in real time by a distributed thermocouple array, and the heating power was dynamically adjusted to keep the temperature fluctuation within ±5 °C. The detection process commenced from room temperature and gradually increased at intervals of 50 °C. After each temperature increase stage, a 5 min thermal equilibrium was maintained before conducting continuous detection (data were collected every 2 min).

The performance verification of the high-temperature sensor encompassed two dimensions: (1) Hardware reliability test: monitoring the thermal stability of the key components (permanent magnets, coils) inside the sensor under different temperature conditions. (2) Defect detection performance evaluation: analyzing the time-domain signal amplitude–temperature correlation of prefabricated defects (including full-penetration circular holes, half-penetration circular holes, and rectangular grooves) to compare the detection capability characteristics of the two types of pipes in a wide temperature range (25–600 °C). The analysis of the experimental results is detailed in Section 4.

## 4. High-Temperature Experiment and Discussion

### 4.1. Stability of Internal Temperature of EMAT at High Temperature

The internal energy exchange of the sensor adhered to the multi-mode heat transfer principle: integrating heat conduction, convective heat transfer, and radiative heat transfer. Among them, the heat exchange between the permanent magnet and the cooling cavity was predominantly governed by conduction, and its heat transfer rate complies with Fourier’s law; was positively correlated with the temperature difference, material thermal conductivity, and contact area; and was negatively correlated with the length of the heat transfer path. To validate the efficacy of the thermal insulation and cooling design, the internal temperature of the sensor was monitored during the detection experiment.

**Figure 15:**
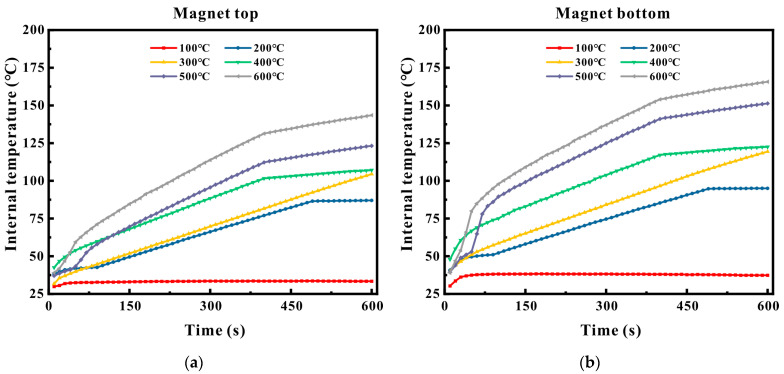
Temperature change inside the sensor: (**a**) magnet tip; (**b**) bottom end of the magnet.

Since the coil was encapsulated in high-temperature ceramic glue, there was no concern about melting and short-circuiting at high temperatures. The primary focus was the thermal stability of the magnet under extremely high temperatures. Due to the large volume of the magnet, a certain temperature gradient existed between the bottom and the top of the magnet. To avoid misjudgment of thermal stability (such as the top not exceeding the maximum operating temperature (350 °C), but the bottom having already exceeded), a thermocouple was placed at the top (the end in contact with water cooling) and the bottom (the end in contact with the coil) of the magnet inside the sensor, and the temperature changes were recorded every 10 s. The results are presented in Figure 15a,b.

As can be observed from Figure 15a, when the surface temperature of the pipe ranged from 100 to 600 °C, the temperature at the top of the magnet consistently remained below 350 °C. When the surface of the pipe was detected at 600 °C for 10 min, the temperature at the top of the magnet was 142 °C. From Figure 15b, it can be noted that the trend of the temperature rise curve at the bottom of the magnet was similar to that at the top, and it also did not exceed the maximum operating temperature. Under different working environment temperatures, the temperature variation at the bottom of the magnet was marked by a relatively steep temperature increase initially, followed by a gradual deceleration and eventual stabilization, reaching a dynamic thermal equilibrium, although the final temperatures differed. When the surface of the pipe was detected at 600 °C for 10 min, the final temperature at the bottom of the magnet was 167 °C.

The experimental outcomes demonstrated that when the pipe wall temperature was no greater than 600 °C and the detection time was no more than 10 min, the temperatures at both the top and bottom of the magnet were lower than 170 °C, significantly beneath the failure critical value of 350 °C. This validates the synergy between the multi-layer insulation structure and the active water-cooling system, enabling the sensor to maintain a certain degree of temperature stability in a high-temperature environment ranging from 500 to 650 °C, thereby ensuring that the signal-to-noise ratio of the detection signal did not fluctuate excessively.

### 4.2. EMAT Defect Detection Capability on High-Temperature Pipes

**Figure 16:**
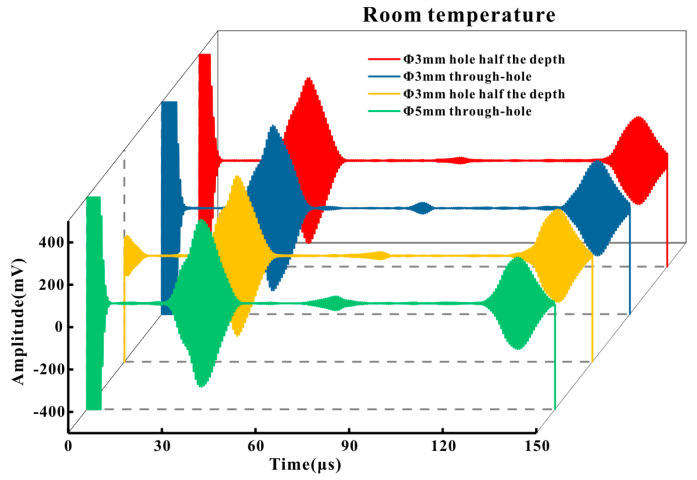
Detection results of different defects of the P91 pipeline at normal temperature.

**Figure 17:**
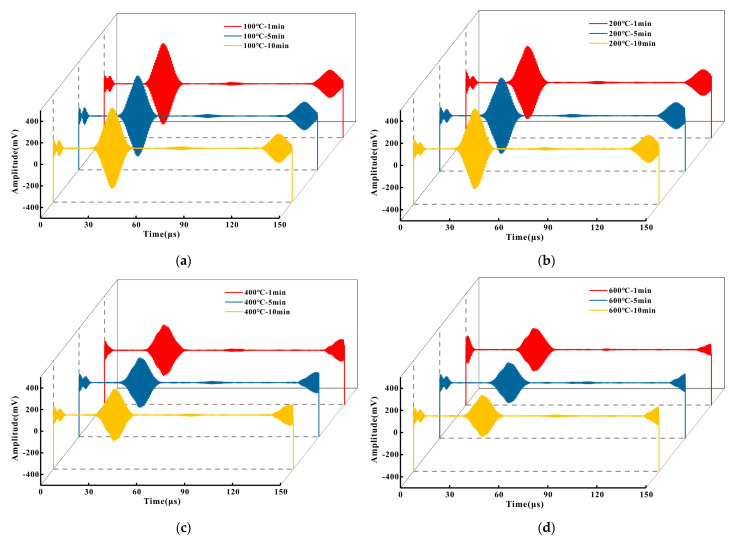
Detection results of the P91 pipe at different contact times under high temperature: (**a**) 100 °C; (**b**) 200 °C; (**c**) 400 °C; (**d**) 600 °C.

**Figure 18:**
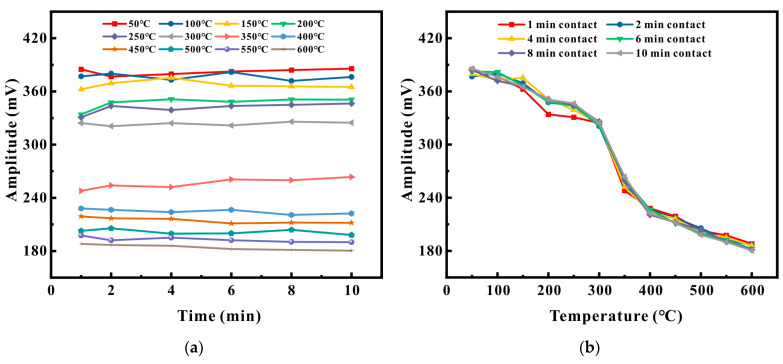
Detection results of the φ3 mm circular semi-hole in the P91 pipe: (**a**) changes in signal amplitude at different temperatures; (**b**) changes in signal amplitude at different detection times.

In order to verify the sensor’s capability of detecting pipeline defects at high temperatures, detections were performed on φ5 and φ3 circular through-holes and φ5 and φ3 circular half-holes on P91 high-temperature pipelines under various temperatures. The results are presented as shown in Figure 16, Figure 17 and Figure 18. Figure 16 depicts the detection outcomes of the four defects at room temperature. From left to right, they are the proximal direct wave, defect echo, and distal direct wave. The defect echo amplitude of the half-hole defect was approximately half of that of the through-hole defect of the same size. Additionally, both increased with the growth of the size. The defect echo amplitude of the 5 mm half-through hole was conspicuously smaller than that of the 3 mm through hole, suggesting that, in contrast to the size of the defect, the depth of the defect exerted a more significant influence on the defect echo amplitude.

Figure 17 presents the signal diagrams for the detection of the 3 mm semi-hole on P91 at 100 °C, 200 °C, 400 °C, and 600 °C. It can be observed from Figure 17a that at 100 °C, although the defect echo amplitude declined slightly compared to that at room temperature, the results remained stable. By comparing the outcomes of detection times of 1 min, 5 min, and 10 min, no significant variations were evident. In Figure 17b, in contrast to Figure 17a, the amplitudes of each wave packet decreased to a certain extent, yet not prominently. Simultaneously, each wave packet exhibited a certain lag, approximately 2.5–3 μs, which is attributed to the increase in temperature. From Figure 17c, it is clearly perceivable that the amplitudes of the wave packets decreased and there was a lag in position. At this point, the amplitude of the direct wave dropped to 61.3% of that at 100 °C, and the amplitude of the defect echo decreased to 66.7% of that at 100 °C. The lag time of the wave packets was approximately 5.2–6.7 μs, but it could still maintain stability within a 10 min detection time. When the pipe temperature reached 600 °C, as depicted in Figure 17d, compared to the detection results at 100 °C, the amplitude reduction and time lag were more pronounced. The amplitude of the direct wave decreased to 49.2% of that at 100 °C, and the amplitude of the defect echo decreased to 51.3% of that at 100 °C. The lag time of the wave packets was approximately 8–9.3 μs. By comparing the results of different detection times, it can be noted that as the detection time extended, the signal amplitude decreased to some degree, but the extent of the decrease was not substantial. By comparing the detection results of different temperatures, it can be seen that the time of direct wave and defect echo appeared later and later. This was caused by a decrease in the CLamb wave speed as the temperature increased. The core reason is that with the increase in temperature, the interatomic binding force was weakened, resulting in a decrease in the elastic modulus, and the wave speed was approximately proportional to the square root of the elastic modulus, so the wave speed slowed down with the decrease in the elastic modulus. Although the thermal expansion of the material at high temperature made the density slightly decrease, and the Poisson ratio slightly increase (theoretically the wave speed should be slightly increased), the change of these parameters led to the increase in the wave speed being far less than the decrease in the elastic modulus, so the overall CLamb wave speed showed a slowing trend under the combined effect.

Figure 18 presents the signal amplitude graph of the φ3 mm circular semi-hole on P91 at various temperatures and different contact times. The variation patterns of the other three defects were analogous. It is conspicuously observable that as the temperature ascended, the amplitude of the defect echo steadily declined, and the descending trend exhibited a state of moderation, intensity, and then moderation. Compared to 50 °C, the amplitude at 600 °C decreased by 53%. It is hypothesized that as the temperature rises, the changes in the electromagnetic parameters related to the material lead to the reduction of the Lorentz force and the magnetostrictive force, causing the signal amplitude to gradually diminish with the increase in temperature. As the detection time extended, it can be discerned that the signal amplitude at each temperature underwent gradual fluctuations, yet the amplitude was not substantial. When the surface temperature of the pipe reached 600 °C, in comparison to 1 min, the detection at 6 min decreased by 1.8%, and the detection result at 10 min decreased by 3.2%. On the whole, it was relatively stable. This substantiates that the sensor is capable of conducting a detection task on the pipe surface with a temperature reaching 600 °C for a maximum scanning duration of 10 min. During this period, the defect signal remained stable, and the minimum detectable defect was a hole with a diameter of φ3 mm and a depth of half the wall thickness.

### 4.3. The Defect Detection Capability of EMAT on Low-Carbon Steel Pipes

**Figure 19:**
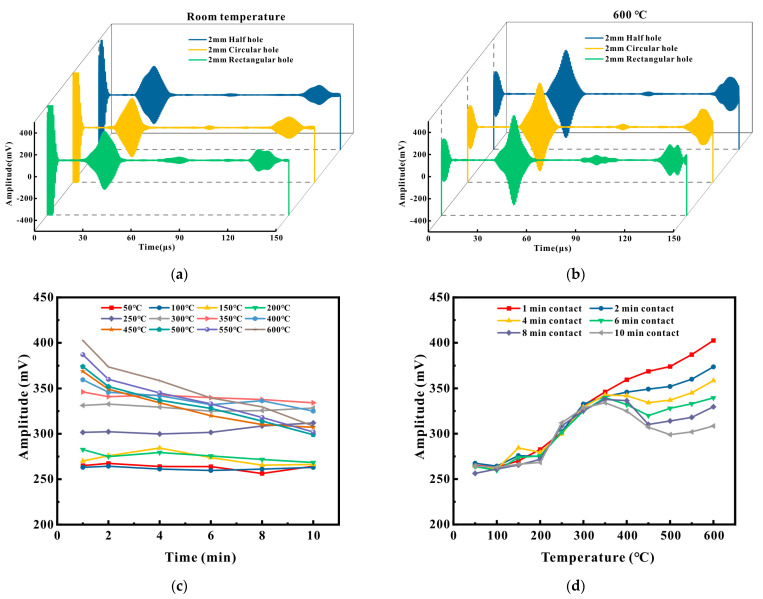
Test results of the Q235 pipeline: (**a**) at normal temperature; (**b**) 600 ° C; (**c**) changes in amplitude of different temperature signals; (**d**) signal amplitude changes at different detection times.

To verify the sensor’s ability to detect defects across supports, detection experiments were conducted on Q235 (a commonly used low-carbon steel) material pipes. The detection results at different temperatures are shown in Figure 19 below.

Figure 19a presents the detection outcomes of EMAT for a 2 mm semi-hole, a 2 mm circular hole, and a 2 mm slot hole at room temperature. From left to right, the four wave packets are the proximal direct wave, the defect echo, and the distal direct wave. It is distinctly observable that the defect echo of the slot hole was the greatest, followed by the circular hole, while the signal of the semi-hole was the least. The results suggest that during detection, for defects of the same size, the slot hole can reflect more waves back compared to the circular hole and was more readily detectable. This is in accordance with the previous experimental findings. Figure 19b depicts the detection results of the three defects at 600 °C. In contrast to Figure 19a, it is evident that the amplitudes of each wave packet increased, and there was a temporal lag. Due to the changes of elastic modulus, density, and Poisson’s ratio at high temperature, the dispersion effect was significantly enhanced. At this time, the velocity reduction of the high-frequency component was much larger than that of the low-frequency component, resulting in different frequency components propagating at different speeds, leading to group velocity separation and phase difference accumulation. At the same time, thermal expansion increased the thickness of the plate, the modal cutoff frequency moved to the low frequency, and the high-frequency signal selectively attenuated because it entered the cutoff region. As a result, the material dispersion curve changed to some extent, but the excitation frequency remained unchanged, resulting in phase distortion and obvious mode separation at high temperature.

Figure 19c,d illustrate the results of detecting a φ2 mm circular through hole on a Q235 pipe under different temperatures and contact times across the support. The variation patterns of the other two defects were similar. From Figure 19c, it can be discerned that as the temperature rose, the signal amplitude also escalated. Beyond a temperature of 350 °C, as the detection time lengthened, the signal amplitude steadily decreased, and the amplitude of the fluctuations became larger. Taking 400 °C, 500 °C, and 600 °C as examples, when the detection time was 2 min, the amplitude change rate at 400 °C was 3.8%, at 500 °C was 5.8%, and at 600 °C was 7.2%. When the detection time was 6 min, the amplitude change rate at 400 °C was 7.6%, at 500 °C was 12.3%, and at 600 °C was 15.7%. When the detection time was 10 min, the amplitude change rate at 400 °C was 9.6%, at 500 °C was 20.1%, and at 600 °C was 24.4%. From Figure 19d, it can be seen that when the contact time was 1 min, as the pipe temperature increased, the signal amplitude gradually augmented, and the relationship between the amplitude and the temperature was proportional. The signal amplitude at 600 °C was approximately 1.52 times that at 50 °C. When the contact time was 2 and 4 min, the signal amplitude still approximately maintained a proportional relationship with the temperature, and the trend was to increase initially, then decrease, and subsequently increase again. The defect echo amplitude at 600 °C remained the greatest. However, when the contact time was 6, 8, and 10 min, the signal amplitude experienced a marked decrease beyond 350–400 °C, and the amplitude at 350–400 °C was the maximum.

Under the same temperature conditions, the amplitude change rate of the Q235 material pipe within the first 6 min at 600 °C was 15.7%, which was significantly higher than the amplitude change rate of 1.8% of the P91 material high-temperature pipe at 600 °C. The amplitude change rate of the Q235 material pipe within the first 10 min at 600 °C was 24.4%, which was considerably higher than the amplitude change rate of 3.2% of the P91 material high-temperature pipe at 630 °C. The signal amplitude of the P91 material pipe was more stable than that of the Q235 material pipe, which may primarily be attributed to the fact that the P91 material pipe (with a maximum operating temperature up to 650 °C) is commonly employed as a high-temperature pipe, and its elastic modulus, Poisson’s ratio, and other parameters exhibit less variation over time at high temperatures, whereas the Q235 material pipe (with a maximum temperature of 350–400 °C) is not well-suited for high-temperature applications. The high-temperature durability performance of the Q235 material pipe is inferior to that of the P91 material pipe, leading to variations in the material parameters of the Q235 material with the duration of heating. Consequently, the higher the temperature, the greater the fluctuation of the detection amplitude over time.

Experimental measurements validate that as the temperature rose within the range of 50–600 °C, the signal amplitude on low-carbon steel underwent a notable enhancement, which is consistent with the prediction in [32]. At high temperatures, due to the increase in temperature, the electromagnetic parameters of magnets and coils undergo significant changes, and the contribution of the Lorentz force mechanism gradually diminishes. Hence, the magnetostrictive mechanism gradually assumes a dominant position. It is hypothesized that the magnetostrictive curve of low-carbon steel undergoes alterations at high temperatures, causing the contribution of the magnetostrictive mechanism to ultrasonic waves to gradually increase with temperature. The signal amplitudes of contact for 1 min and 10 min, as well as those at 50 °C and 600 °C, can substantiate this point.

## 5. Summary

A large magnet lift-off circumferential guided wave sensor for defect detection in inaccessible areas of high-temperature pipelines was developed through simulations and experiments, and its validity was verified via experiments. The conclusions drawn are as follows:

Based on the excitation principle of EMAT, a two-dimensional simulation model for pipeline detection was established. It was discovered that Halbach magnets can offer a stronger magnetic field compared to conventional magnets. Their performance in the detection of ferromagnetic materials (low-carbon steel) with small lift-offs was not as good as that of conventional magnets, but they exhibited superior performance at large magnet lift-offs. Verified through experiments, when the magnet lift-off was 9 mm, the signal amplitude of Halbach magnets was approximately twice that of conventional magnets, which undoubtedly proves highly beneficial for the sensor to avoid overheating at high temperatures. Based on the experimental outcomes, a high-temperature EMAT was designed.

By conducting real-time monitoring of the internal temperature of the transducer during the surface detection process of the pipeline at high temperatures, it was found that the combined action of a sufficiently large magnet lift-off and a water-cooling system can maintain the internal temperature of the sensor below 200 °C. This indicates that the sensor can accomplish the fault detection task within a certain period at high temperatures without the risk of thermal damage.

At 600 °C, EMAT is capable of detecting defects in P91 pipes with a minimum diameter of φ3 mm and a depth of half the wall thickness. After continuous detection for 10 min, there was no significant signal attenuation. In the experiment, it was observed that the amplitude of the defect return wave of P91 high-temperature pipes gradually decreased as the temperature rose, and the signal amplitude at 600 °C was approximately 53% lower than that at 50 °C.

On a Q235 pipe at 600 °C, EMAT successfully detected a 2 mm semi-hole. The test results of low-carbon steel indicate that when the contact time was short, the amplitude of the defect echo was approximately 1.5 times higher than that under normal temperature. However, after the contact time exceeded 5 min, it was found that the maximum value of the signal occurred between 350 and 400 °C, which might be related to the maximum working temperature of Q235 (constrained by the properties of the material itself).

## Figures and Tables

**Table 1:** Simulation parameters.

Name	Low-Carbon Steel	Copper
Density (kg/m^3^)	7726	/
Young’s modulus (GPa)	210	/
Poisson’s ratio	0.30	/
Relative dielectric constant	1	1
Electric conductivity (MS/m)	6.65	58
Relative permeability	130	1

## Data Availability

Data are contained within the article.
